# Functional connectivity in resting‐state networks relates to short‐term global cognitive functioning in cardiac arrest survivors

**DOI:** 10.1002/hbm.26769

**Published:** 2024-10-24

**Authors:** Marlous M. L. H. Verhulst, Hanneke M. Keijzer, Pauline C. W. van Gils, Caroline M. van Heugten, Frederick J. A. Meijer, Bart A. R. Tonino, Judith L. Bonnes, Thijs S. R. Delnoij, Jeannette Hofmeijer, Rick C. Helmich

**Affiliations:** ^1^ Clinical Neurophysiology, TechMed Centre University of Twente Enschede The Netherlands; ^2^ Department of Neurology Rijnstate Hospital Arnhem The Netherlands; ^3^ Department of Psychiatry and Neuropsychology, School for Mental Health and Neuroscience Maastricht University Maastricht The Netherlands; ^4^ Limburg Brain Injury Center Maastricht University Maastricht The Netherlands; ^5^ Department of Neuropsychology and Psychopharmacology Maastricht University Maastricht The Netherlands; ^6^ Department of Medical Imaging Radboud University Medical Center Nijmegen The Netherlands; ^7^ Department of Radiology Rijnstate Hospital Arnhem The Netherlands; ^8^ Department of Cardiology Radboud University Medical Center Nijmegen The Netherlands; ^9^ Department of Cardiology Maastricht University Medical Center+ Maastricht The Netherlands; ^10^ Donders Institute for Brain, Cognition and Behaviour, Centre of Expertise for Parkinson and Movement Disorders, Neurology Department Radboud University Medical Centre Nijmegen The Netherlands; ^11^ Donders Institute for Brain, Cognition and Behaviour, Centre for Cognitive Neuroimaging Radboud University Nijmegen The Netherlands

**Keywords:** cardiac arrest, cognition, functional connectivity, functional MRI, resting‐state networks

## Abstract

Long‐term cognitive impairment is common in cardiac arrest survivors. Screening to identify patients at risk is recommended. Functional magnetic resonance brain imaging (fMRI) holds potential to contribute to prediction of cognitive outcomes. In this study, we investigated the possible value of early changes in resting‐state networks for predicting short and long‐term cognitive functioning of cardiac arrest survivors. We performed a prospective multicenter cohort study in cardiac arrest survivors in three Dutch hospitals. Resting‐state fMRI scans were acquired within a month after cardiac arrest. We primarily focused on functional connectivity within the default‐mode network (DMN) and salience network (SN), and additionally explored functional connectivity in seven other networks. Cognitive outcome was measured using the Montreal Cognitive Assessment (MoCA) during hospital admission and at 3 and 12 months, and by neuropsychological examination (NPE) at 12 months. We tested mixed effects models to evaluate the value of connectivity within the networks for predicting global cognitive outcomes at the three time points, and long‐term cognitive outcomes in the memory, attention, and executive functioning domains. We included 80 patients (age 60 ± 11 years, 72 (90%) male). MoCA scores increased significantly between hospital admission and 3 months (ΔMoCA_hospital‐3M_ = 2.89, *p* < 0.01), but not between 3 and 12 months (ΔMoCA_3M–12M_ = 0.38, *p* = 0.52). Connectivity within the DMN, SN, and dorsal attention network (DAN) was positively related to global cognitive functioning during hospital admission (*β*_DMN_ = 0.85, *p* = 0.03; *β*_SN_ = 1.48, *p* < 0.01; *β*_DAN_ = 0.96, *p* = 0.01), but not at 3 and 12 months. Network connectivity was also unrelated to long‐term memory, attention, or executive functioning. Resting‐state functional connectivity in the DMN, SN, and DAN measured in the first month after cardiac arrest is related to short‐term global, but not long‐term global or domain‐specific cognitive performance of survivors. These results do not support the value of functional connectivity within these RSNs for prediction of long‐term cognitive performance after cardiac arrest.

## Graphical abstract

Higher resting‐state functional connectivity within the default‐mode network and salience network measured within the first weeks after cardiac arrest is related to better short‐term, but not long‐term cognitive functioning of cardiac arrest survivors.

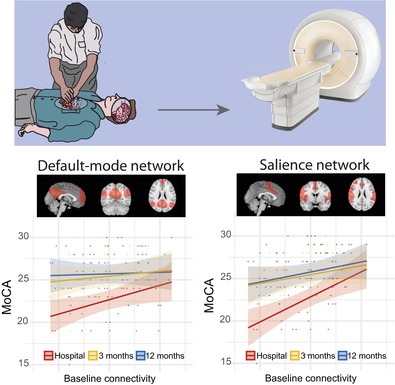

## 1 INTRODUCTION

The global incidence of out‐of‐hospital cardiac arrest is 20–186 per 100,000 people, yearly (Berdowski et al., 2010). Survival rates up to 1 year have significantly increased, from 8% in 2000–2009 to over 13% in 2010–2019 (Yan et al., 2020). Increased survival rates indicate an increase in the number of patients living with the consequences of cardiac arrest. Cognitive impairments are present in approximately half of the cardiac arrest survivors, most frequently in the memory, attention, and executive functioning domains (Moulaert et al., 2009). Cognitive impairment is strongly related to lower participation and quality of life (Lim et al., 2014; Moulaert et al., 2010; Ørbo et al., 2015).

Current guidelines recommend assessment of physical and non‐physical impairments prior to hospital discharge to determine early rehabilitation needs (Nolan et al., 2021). Furthermore, it is recommended to estimate the risk of long‐term cognitive and emotional problems (Nolan et al., 2021). However, it is unclear how impairments should be assessed before discharge and which measures can help identify patients at risk for enduring impairments.

Studies on early identification of patients with long‐term cognitive impairment after cardiac arrest are scarce. There is some evidence for a longer coma duration (Sauvé, Doolittle, et al., 1996; Sauvé, Walker, et al., 1996), reduced left ventricular function (Sauvé, Doolittle, et al., 1996), elevated S‐100B concentration in blood (Prohl et al., 2007; Prohl et al., 2009), and a score below the threshold on a bedside cognitive screening tool (Prohl et al., 2007, 2009) being predictive. However, the evidence is weak because of small sample sizes and lack of external validation of the identified possible predictors (Glimmerveen et al., 2023).

Cerebral magnetic resonance imaging (MRI) can provide insight into the nature and severity of brain damage and holds the potential to contribute to predicting long‐term outcomes, including cognitive functioning. Previous studies have shown that MRI markers, such as diffusion metrics or changes in functional connectivity in resting‐state networks, hold relevant predictive value for gross neurological outcome of comatose cardiac arrest patients (Hirsch et al., 2015; Keijzer, Lange, et al., 2022; Keijzer, Verhulst, et al., 2022; Koenig et al., 2014; Norton et al., 2012; Sair et al., 2018; Wagner et al., 2020; Wijman et al., 2009), as well as cognitive outcomes in patients after acute onset brain injury such as stroke and traumatic brain injury (Verhulst et al., 2023). However, these MRI markers have not been studied in relation to cognitive outcomes in cardiac arrest survivors. Furthermore, larger volumes of global grey matter, amygdala, hippocampus, or temporal lobe have been related to better memory performance, while larger white matter volume was related to worse performance in cardiac arrest survivors (Allen et al., 2006; Grubb et al., 2000; Ørbo et al., 2018; Stamenova et al., 2018). However, these results were obtained from cross‐sectional studies, based on simultaneous MRI scans and cognitive testing at least 3 months after cardiac arrest, and therefore provide no information on the value of early MRI for prediction of long‐term impairments.

Acute brain damage in cardiac arrest survivors may involve both functional and structural changes. For instance, diffusion‐weighted imaging (DWI) and fluid‐attenuated inverse recovery (FLAIR) scans in the first days after cardiac arrest may show no visible damage, even despite enduring cognitive impairment (Keijzer, Verhulst, et al., 2022), hinting at functional changes. A resting‐state network analysis might provide useful insight into these functional changes in relation to cognitive functioning. Two resting‐state networks that play an important role in cognitive functioning are the default‐mode network (DMN) (Mak et al., 2017; Raichle, 2015) and salience network (SN) (Uddin, 2017). The DMN, especially the posterior cingulate cortex, has a hub‐function in the brain and plays an important role in mediating information flow across various brain circuits (de Pasquale & Marzetti, 2019; Hagmann et al., 2008; Leech & Sharp, 2014). Previous research on resting‐state networks and cognitive outcome after acute onset brain injury showed that higher functional connectivity in the DMN was related to better cognitive outcome (Verhulst et al., 2023). Additionally, decreases in grey matter density in the precuneus and posterior cingulate cortex (Mueller et al., 2020) and decreases in precuneus connectivity (Mueller et al., 2023) were seen in patients with heart failure. Atrophy of the precuneus at least 3 months after cardiac arrest was related to worse memory performance (Horstmann et al., 2010), while decoupling of the precuneus was associated with worse executive functioning in patients with heart failure (Schroeter et al., 2023). Finally, our group has previously shown that functional connectivity within the DMN and SN distinguished comatose patients with good from those with poor gross neurological outcomes after cardiac arrest (Keijzer, Lange, et al., 2022).

With the current analyses, we aim to study the relation between functional connectivity within the DMN and SN and cognitive functioning, as well as the possible value of early changes in those networks for predicting long‐term cognitive functioning in patients after cardiac arrest surviving up to recovery of consciousness. Based on previous findings (Keijzer, Lange, et al., 2022), we expect that reduced functional connectivity in the DMN and SN at baseline is related to worse cognitive performance during follow‐up. The results will provide input to our ongoing efforts to determine the value of MRI for identifying patients at risk for enduring long‐term cognitive impairments after cardiac arrest.

## 2 MATERIALS AND METHODS

### 2.1 Study design

We performed a multicenter prospective longitudinal cohort study: the Brain Outcome after Cardiac Arrest‐prediction (BROCA‐prediction) study. The study was designed to establish a multimodal prediction model for cognitive, emotional, and participation outcomes of cardiac arrest survivors (Netherlands Trial Register NL9451). For current analyses, we added data of patients with brain MRI scans within 10 days and neuropsychological outcome at 12 months from the multicenter prospective Cracking Coma study (clinicaltrials.gov NCT03308305), which was designed to establish a multimodal prediction model for the gross neurological outcome of comatose cardiac arrest patients and largely overlaps with BROCA‐prediction. These data can be combined because MRI protocols and neuropsychological assessments are equal.

We used MRI data collected within the first month after cardiac arrest and cognitive outcome data collected within the first month (during hospital admission) and at 3 and 12 months after cardiac arrest from patients included between November 2019 and October 2022 in the BROCA‐prediction study. Data were collected in Rijnstate (Arnhem, The Netherlands), Radboud University Medical Center (Nijmegen, The Netherlands), and Maastricht University Medical Center+ (Maastricht, The Netherlands). Study protocols were approved by the Committee on Research Involving Human Subjects region Arnhem‐Nijmegen (NL69767.091.19, NL62151.091.17).

### 2.2 Study population

Adult cardiac arrest patients (age ≥ 18 years) were included in the studies during hospital admission. Pregnancy, a known progressive neurological disease, and pre‐existing dependency in daily living were exclusion criteria. Patients who died in the ICU before recovery of consciousness were not included in the current analyses. Written consent was given by the patient or legal representative (in case of patient incapacity). Initial consent given by a legal representative was confirmed by the patient after recovery of capacity.

### 2.3 Treatment

All patients were treated according to international guidelines (Nolan et al., 2021) and local protocols. For ICU treatment, this included targeted temperature treatment at 36 or 32–34°C (center dependent) followed by passive rewarming and active normothermia maintenance. For the patients surviving up to hospital discharge, treatment included standardized cardiac rehabilitation programs. MRI scans were not considered in treatment decisions.

### 2.4 Outcome measures

Outcome was assessed using the Montreal Cognitive Assessment (MoCA) and a neuropsychological test battery.

The MoCA is a widely used screening instrument for cognitive functioning (Nasreddine et al., 2005) validated in the cardiac arrest population (Van Gils et al., 2022). Global cognitive functioning based on the MoCA was assessed during hospital admission and at 3 and 12 months after cardiac arrest. Scores range from 0 to 30 with a correction for educational level, by giving those with 12 or fewer years of formal education an extra point. A score of ≥26 is considered normal (Nasreddine et al., 2005).

Memory, attention, and executive functioning at 12 months after cardiac arrest were assessed using the Rey Auditory Verbal Learning Test (RAVLT; memory), Trail Making Test A and B (TMT‐AB; attention, executive functioning), Stroop Color and Word Test (Stroop; attention, executive functioning), short Raven's progressive matrices test (short Raven; executive functioning), and letter fluency (executive functioning). *Z*‐scores corrected for age, sex, and educational level were calculated using Maasnorms (Diesfeldt, 2006) for the RAVLT, TMT‐AB, and Stroop, using NIP norms (Schmand et al., 2012) for letter fluency, and based on previous research (Hunter et al., 2022) for the short Raven. *Z*‐scores outside the range of −3.00 to +3.00 were adjusted to −3.00 or +3.00, respectively. A composite *z*‐score for each cognitive domain (memory, attention, and executive functioning) was calculated based on the mean of all *z*‐scores of the subtests within the specific domain (Table S1). A composite *z*‐score of −1.5 or lower indicated impairment in that domain (Sachdev et al., 2014; Schellekens et al., 2023).

### 2.5 MRI data acquisition

All patients underwent 3 T MRI scanning on Philips Ingenia (Rijnstate, Maastricht UMC+) or Siemens Skyra (Radboudumc) scanners during hospital admission within 1 month after cardiac arrest.

Anatomical data were acquired using 3D‐T1 (Philips Rijnstate: 3D TFE, TR/TE 3.8/8300 ms, voxel size 1.0*1.0*1.0 mm; Philips Maastricht UMC+: cs 3D ISO TR/TE 6.57/2.95 ms, voxel size 1.0*1.0*1.0 mm; Siemens Radboudumc: MPRAGE TR/TE 3.41/2400 ms, voxel size 0.9*0.9*1.0 mm). Resting‐state fMRI was acquired using a gradient‐echo echo planar imaging sequence (Philips: TE/TR 27/2220 ms, voxel size 3.0*3.0*3.0 mm, 220 volumes; Siemens: TE/TR 27/2280 ms, voxel size 3.2*3.2*3.0 mm, 220 volumes).

### 2.6 MRI data analysis

Quality assessment of the T1‐weighted and rsfMRI scans was first performed visually. MRIqc (Esteban et al., 2017) was used for further quality evaluation.

MRI pre‐processing was done using fMRIPrep 21.0.1 (Esteban et al., 2019), which is based on Nipype 1.6.1 (Gorgolewski et al., 2011, 2018) (RRID:SCR_002502). This pipeline includes motion correction, resampling to MNI152Lin6cAsym standard space, removal of non‐steady state volumes, spatial smoothing with an isotropic, Gaussian kernel of 6 mm full‐width half‐maximum, and non‐aggressive denoising based on independent component analysis (ICA‐AROMA) (Pruim et al., 2015). More information on the preprocessing steps in the fMRIPrep pipeline can be found in Data S1. Data quality of fMRIPrep output was visually checked. Subsequently, additional smoothing to reach a net 8 mm full‐width half‐maximum, removal of first five volumes, and high pass filtering (0.007 Hz) were performed. The data quality of the output was visually rechecked.

We performed probabilistic independent component analysis (ICA) on a group level using FSL's MELODIC (Beckmann & Smith, 2004) based on the MRI data of all included patients, resulting in 20 group‐average spatially independent components. Each of these 20 components were visually classified as a resting‐state network or noise.

We regressed the non‐noise group‐average spatial components to the individual maps using FSL's Dual Regression (Beckmann et al., 2009). Study site, mean centered framewise displacement, and conscious state during scanning (comatose or conscious) were added as covariates. The resulting maps of the identified resting‐state networks represent voxel‐wise connectivity strength within the respective network for each individual patient. We used the spatial maps of the DMN and SN for our primary analyses. In addition, we explored effects in seven other networks (lateral visual network [VNL], medial visual network [VNM], somatomotor network [SMN], executive control network [ECN], cerebellar network [CBN], dorsal attention network [DAN], and frontoparietal network [FPN]). Mean normalized functional connectivity strength (*z*‐normalized) within each of the resting‐state networks was calculated for each patient and related to clinical variables, as outlined below. A separate group ICA analysis using FSL's MELODIC was performed on the patients who were comatose during the MRI to evaluate the integrity of the DMN in this group.

### 2.7 Statistical analysis

Data are presented as mean and standard deviations (SD) or median and interquartile ranges (IQR). We used chi‐square tests for ordinal variables and unpaired *t* tests or Mann–Whitney *U* tests for continuous variables to compare groups or differences from zero.

A mixed effects ANOVA was performed to assess the change in MoCA scores over time. Multimodal mixed effects regression models with random intercept were created to assess the relation between mean connectivity strength within the resting‐state networks and cognitive functioning. Outcome measures were the MoCA scores at the three different time points (hospital admission, 3 months, and 12 months after cardiac arrest). Fixed effects were study site, conscious state during MRI, and time between cardiac arrest and MRI. An interaction between mean connectivity strength and time point (hospital admission, 3 months, and 12 months) was added as a fixed effect to evaluate the relationship between connectivity strength and MoCA scores at these three time points and to assess the change in relationship over time. Patient ID was used as a random effect. Additionally, a mixed effects regression model was created without the interaction with time point to assess the overall relationship between mean connectivity strength and MoCA scores.

Similar multimodal mixed effects regression models with random intercept were created to assess the predictive value of mean connectivity strength within the resting‐state networks for *z*‐scores of the neuropsychological examination at 12 months. Here, an interaction between mean connectivity strength and cognitive domain (memory, attention, and executive functioning) was added as a fixed effect. Additionally, a mixed effects regression model was created without the interaction with cognitive domain to assess the overall relationship between mean connectivity strength and neuropsychological scores. Predictive values of the models were evaluated using regression coefficients.

The relationship between connectivity in the resting‐state networks and cognitive impairment was assessed by logistic multimodal mixed effects models with dichotomized outcomes based on the thresholds for MoCA and NPE (score <26 and *Z* < −1.5, respectively). Predictive values of these models were evaluated using odds ratios.

A sensitivity analysis was performed to assess the influence of conscious state during the MRI by repeating the mixed effects analyses for DMN and SN after exclusion of the 12 patients who were comatose during MRI scanning.

Voxel wise relationships between network strength in the DMN and SN and cognitive outcome were explored by performing a dual regression on the network maps (*z*‐normalized) with the MoCA at all three timepoints. Analyses were performed using threshold‐free cluster enhancement and corrected with 5% family‐wise error (FWE). Study site, comatose state during the MRI, and mean centered framewise displacement were added as covariates.

Mean connectivity in the DMN was compared between patients who were comatose and patients who were conscious during MRI scanning using a Mann–Whitney *U* test.

*P*‐values <0.05 were assumed statistically significant. Statistical analyses were performed using R version 4.0.0.

## 3 RESULTS

### 3.1 Patient characteristics

Baseline characteristics of the patients (*n* = 80) are presented in Table 1. The mean age was 60 ± 11 years, and the majority of patients were men (90%) and had a shockable initial heart rhythm (98%) (Cho et al., 2022; Shuvy et al., 2017).

**TABLE 1:** Clinical and demographic characteristics of the study population.

Characteristic	*N* = 80
Male	72 (90%)
Age (years)	60 ± 11
Time to ROSC (min)	10 [3–63]
Initial heart rhythm	
Shockable	78 (98%)
Non‐shockable	2 (2%)
Comatose on hospital admission	64 (80%)
Comatose during MRI scan	12 (15%)
Time from cardiac arrest to MRI (days)	4.5 [1.5–35]
MoCA during hospital admission (*n* = 54)	24 [7–29]
Score <26	36 (67%)
MoCA at 3 months (*n* = 47)	26 [19–30]
Score <26	20 (43%)
MoCA at 12 months (*n* = 62)	26 [10–30]
Score <26	31 (50%)
Memory component *z*‐score (*n* = 58)	−0.86 [−2.88–1.85]
Score <−1.5	14 (24%)
Attention component *z*‐score (*n* = 58)	−0.65 [−3.00–0.76]
Score <−1.5	18 (31%)
Executive functioning component *z*‐score (*n* = 60)	−0.30 [−3.00–1.45]
Score <−1.5	7 (12%)

*Note*: Data are represented as number (%), mean ± standard deviation, or median [range].

Abbreviations: MoCA, Montreal Cognitive Assessment; ROSC, return of spontaneous circulation.

### 3.2 Evolution of cognitive outcome

**FIGURE 1:**
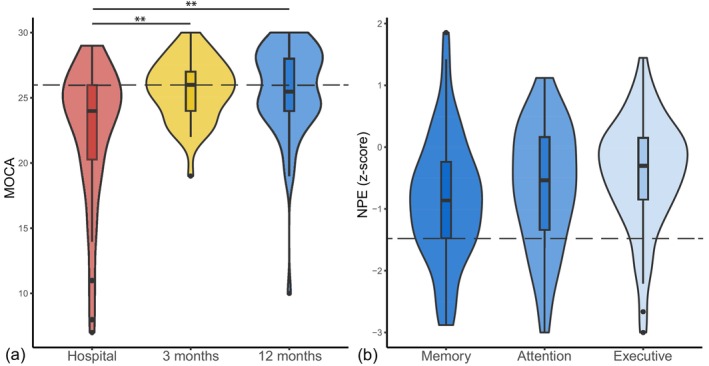
(a) Overview of MoCA scores during hospital admission (red, left), 3 months (yellow, middle), and 12 months (blue, right) after cardiac arrest. (b) Overview of memory scores (dark blue, left), attention scores (blue, middle), and executive functioning scores (light blue, right) at 12 months after cardiac arrest. Violins show density of the cognitive scores. Asterisks indicate levels of significance (*p < 0.05, **p < 0.01), dashed lines indicate the thresholds for cognitive impairment (MoCA <26, NPE *z*‐score <−1.5). MoCA = Montreal Cognitive Assessment; NPE, neuropsychological examination.

During hospital admission, 67% of patients had cognitive impairment defined as a MoCA score <26. This number decreased to 43% and 50% at 3 and 12 months, respectively. The distribution of MoCA scores at the three time points is shown in Figure 1a. The MoCA score significantly increased on a group‐level between hospital admission and 3 months (ΔMoCA_hospital‐3M_ = 2.89, *p* < 0.01), but not between 3 and 12 months (ΔMoCA_3M–12M_ = 0.38, *p* = 0.52).

At 12 months, many patients still had cognitive impairment as measured with a full NPE, mostly in the domains of attention (31% with *z* < −1.5), followed by impairment of memory (26%), and impairment of executive function (12%). The distribution of component scores for the three cognitive domains is shown in Figure 1b. On a group level, memory, attention, and executive function component scores were significantly lower than zero.

### 3.3 Relation between functional connectivity and cognitive functioning

**FIGURE 2:**
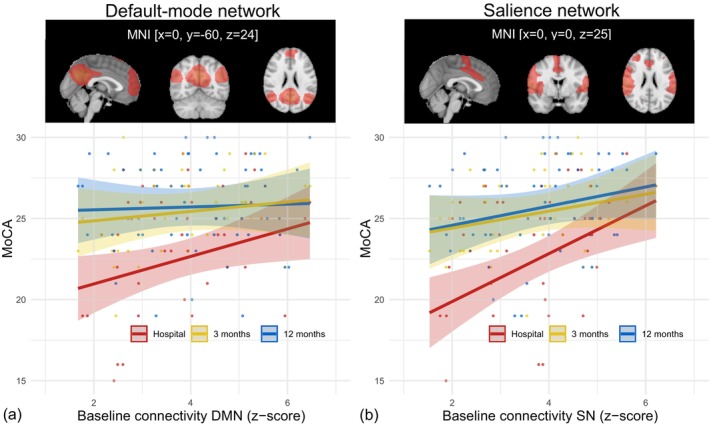
Top panels: Spatial maps of the two resting‐state networks. Lower panels: Results of mixed effects regression models of connectivity strength within the default‐mode network (a) and salience network (b) with MoCA scores during hospital admission, 3 months, and 12 months after cardiac arrest. These results show a positive relation between connectivity strength in DMN or SN and MoCA score during hospital admission and, but not between connectivity strength and MoCA score at 3 and 12 months after cardiac arrest. Results are corrected for study site, conscious state during MRI, and time between cardiac arrest and MRI. DMN, default‐mode network; MoCA, Montreal Cognitive Assessment; SN, salience network.

A group average of the DMN and SN images is shown in Figure 2 (top panel). Statistics are shown in Table 2 (DMN) and Table 3 (SN). For the DMN, we found no significant interaction between network connectivity and timing of the MoCA score. Connectivity within the DMN was positively related to MoCA score during hospital admission (*β* = 0.85, *p* = 0.03), but not with MoCA scores at 3 months or 12 months.

**TABLE 2:** Results of mixed effects regression model of connectivity strength within the default‐mode network with MoCA scores during hospital admission, 3 months, and 12 months after cardiac arrest.

Predictors	MoCA		MoCA hospital		MoCA 3 months		MoCA 12 months	
	Estimates	*p*‐value	Estimates	*p*‐value	Estimates	*p*‐value	Estimates	*p*‐value
Intercept	23.49 [20.97, 26.00]	<0.01	19.86 [16.67, 23.06]	<0.01	24.87 [21.50, 28.24]	<0.01	25.95 [22.78, 29.11]	<0.01
DMN	0.45 [−0.12, 1.02]	0.12	**0.85 [0.10, 1.60]**	**0.03**	0.29 [−0.49, 1.07]	0.47	0.09 [−0.64, 0.82]	0.81
Δ hospital					0.56 [−0.25, 1.37]	0.18	0.76 [−0.07, 1.59]	0.07
Δ 3 months			−0.56 [−1.37, 0.25]	0.18			0.20 [−0.65, 1.05]	0.64
Δ 12 months			−0.76 [−1.59, 0.07]	0.07	−0.20 [−1.05, 0.65]	0.64		
Study site								
RAD	1.35 [−0.91, 3.61]	0.24	0.51 [−1.76, 2.78]	0.66	0.51 [−1.76, 2.78]	0.66	0.51 [−1.76, 2.78]	0.66
MUMC	1.58 [−0.60, 3.76]	0.16	1.48 [−0.80, 3.75]	0.20	1.48 [−0.80, 3.75]	0.20	1.48 [−0.80, 3.75]	0.20
Comatose	**−2.52 [−4.70, −0.34]**	**0.02**	**−2.50 [−4.69, −0.31]**	**0.03**	**−2.50 [−4.69, −0.31]**	**0.03**	**−2.50 [−4.69, −0.31]**	**0.03**
MRI time	−0.10 [−0.20, 0.01]	0.06	−0.08 [−0.18, 0.03]	0.16	−0.08 [−0.18, 0.03]	0.16	−0.08 [−0.18, 0.03]	0.16

*Note*: This table shows the estimate with 95% confidence interval and *p*‐value. Bold type font indicates statistical significance (*p* < 0.05).

Abbreviation: DMN, default‐mode network.

**TABLE 3:** Results of mixed effects regression model of connectivity strength within the salience network with MoCA scores during hospital admission, 3 months, and 12 months after cardiac arrest.

Predictors	MoCA		MoCA hospital		MoCA 3 months		MoCA 12 months	
	Estimates	*p*‐value	Estimates	*p*‐value	Estimates	*p*‐value	Estimates	*p*‐value
Intercept	22.13 [19.57, 24.69]	<0.01	17.63 [14.27, 21.00]	<0.01	24.07 [20.59, 27.55]	<0.01	24.11 [20.93, 27.29]	<0.01
SN	**0.85 [0.23, 1.47]**	**<0.01**	**1.48 [0.64, 2.31]**	**<0.01**	0.52 [−0.34, 1.38]	0.23	0.59 [−0.18, 1.36]	0.13
Δ hospital					**0.95 [0.07, 1.84]**	**0.03**	**0.89 [0.01, 1.76]**	**0.05**
Δ 3 months			**−0.95 [−1.84, −0.07]**	**0.03**			−0.07 [−0.96, 0.83]	0.88
Δ 12 months			**−0.89 [−1.76, −0.01]**	**0.05**	0.07 [−0.83, 0.96]	0.88		
Study site								
RAD	1.19 [−1.01, 3.39]	0.29	0.31 [−1.90, 2.53]	0.78	0.31 [−1.90, 2.53]	0.78	0.31 [−1.90, 2.53]	0.78
MUMC	1.67 [−0.42, 3.75]	0.12	1.59 [−0.61, 3.79]	0.16	1.59 [−0.61, 3.79]	0.16	1.59 [−0.61, 3.79]	0.16
Comatose	−2.06 [−4.21, 0.08]	0.06	−2.11 [−4.28, 0.06]	0.06	−2.11 [−4.28, 0.06]	0.06	−2.11 [−4.28, 0.06]	0.06
MRI time	**−0.12 [−0.22, −0.02]**	**0.02**	−0.09 [−0.20, 0.01]	0.09	−0.09 [−0.20, 0.01]	0.09	−0.09 [−0.20, 0.01]	0.09

*Note*: This table shows the estimate with 95% confidence interval and *p*‐value. Bold type font indicates statistical significance (*p* < 0.05).

Abbreviation: SN, salience network.

For the SN, we found that the slope of the relationship between network connectivity and MoCA score was significantly steeper during hospital admission than at 3 months (Δ*β*_hospital‐3M_ = 0.95 (95%CI: 0.07–1.84), *p* = 0.03) and 12 months (Δ*β*_hospital‐12M_ = 0.89 (95%CI: 0.01–1.76), *p* = 0.05). Connectivity within the SN was positively related to MoCA scores during hospital admission (*β* = 1.48, *p* < 0.01), but not with MoCA scores at 3 or 12 months.

Patients who were comatose during MRI scanning had lower MoCA scores than patients who were conscious (significant effect of “Comatose” covariate in the first model, *p* = 0.03). Study site and time between cardiac arrest and MRI did not significantly influence the MoCA scores (Figure 2 and Tables 2 and 3).

In line with our finding that network connectivity was not related to MoCA scores at 12 months, we observed no relation between network connectivity in DMN or SN and cognitive performance in the subdomains memory, attention, or executive functioning, derived from full NPE at 12 months (Tables S2 and S3).

Results from our sensitivity analysis, where we excluded the 12 patients who were comatose during MRI scanning, were essentially similar to analyses based on the whole group (Tables S4–S7).

In our exploratory analyses, we found that network connectivity in the DAN was positively related to MoCA scores during hospital admission. In the other six networks (VNL, VNM, SMN, ECN, CBN, and FPN) we found no relationship between network connectivity and MoCA scores (Table S8). Additionally, we observed no relationship between network connectivity in any of the networks and cognitive performance in the subdomains memory, attention, or executive functioning, derived from full NPE at 12 months (Table S9).

In our voxel‐wise analyses, we found that higher connectivity in the right precuneus (DMN) and right anterior temporoparietal junction (SN) were related to better MoCA scores during hospital admission (Figure S1).

### 3.4 Relation between functional connectivity and cognitive impairment

When dichotomizing between normal cognition and cognitive impairment (MoCA score < 26 or NPE *z*‐score < −1.5), we did not find a relationship between connectivity within the DMN or SN and cognitive impairment during hospital admission or at 3 or 12 months based on the MoCA. Similarly, connectivity strength was unrelated to cognitive impairment in the memory, attention, or executive functioning domain, based on neuropsychological tests at 12 months in our analyses.

### 3.5 DMN integrity in comatose patients

Mean connectivity in the DMN did not differ between patients who were comatose (connectivity = 3.09 [IQR 2.34–5.02]) and patients who were conscious (connectivity = 3.99 [IQR 3.23–5.06]) during MRI scanning (*p* = 0.22). The DMN could be identified in a group ICA analysis of the 12 patients who were comatose during MRI scanning (Figure S2).

## 4 DISCUSSION

We found a clear relationship between mean connectivity strength within the DMN, SN, and DAN and short‐term global cognitive functioning of survivors in the first weeks after cardiac arrest. Stronger connectivity was related to better global cognitive functioning. The strength of this relationship between network connectivity and cognitive functioning became smaller with follow‐up and was no longer significant at 3 and 12 months. In line with the fact that we did not find a correlation between connectivity strength and cognitive function at 12 months, we also did not observe a relationship between functional connectivity and cognitive functioning in the subdomains of memory, attention, and executive dysfunction derived from a full NPE at 12 months. These findings indicate that the strength of these resting‐state networks reflects the degree of brain functioning during hospital admission, but cannot serve as a predictive measure for long‐term cognitive functioning after cardiac arrest.

In our sample, indications for cognitive impairments based on screening were present in 69% of patients during hospital admission and 43%–50% at follow‐up. It might be argued that our sample has relatively mild injury from cardiac arrest, since all patients could undergo MRI scanning and the majority was already conscious during the scan. However, the percentages of patients showing indications for cognitive impairments are consistent with prior research, indicating that approximately half of patients experience long‐term cognitive impairment after cardiac arrest (Green et al., 2015; Hagberg et al., 2022; Moulaert et al., 2009). MoCA scores significantly increased between hospital admission and 3 months, but showed no significant change between 3 and 12 months. This emphasizes the previous finding that the recovery of cognitive functioning occurs predominantly within the first 3 months after cardiac arrest and stabilizes in the period thereafter (Moulaert et al., 2017). Therefore, we believe that our study sample is a good representation of patients who survive cardiac arrest.

To the best of our knowledge, this is the first study examining the relationship between resting‐state networks and cognitive outcomes after cardiac arrest. Previous research showed that resting‐state network strength, specifically in the DMN, might contribute to the prediction of gross neurological outcomes of comatose patients after cardiac arrest on the ICU (Keijzer, Lange, et al., 2022; Koenig et al., 2014; Norton et al., 2012; Sair et al., 2018; Wagner et al., 2020). One study performing resting‐state fMRI in cardiac arrest survivors showed that functional activity, assessed using amplitude of low‐frequency fluctuations and regional homogeneity, was altered in cardiac arrest survivors compared to healthy controls (Wu et al., 2023). However, none of these studies provides information on the value of early fMRI for predicting long‐term cognitive outcomes.

Studies on the relation between connectivity in resting‐state networks and cognitive performance in other populations with acute onset brain injury showed that lower connectivity in the DMN was significantly associated with worse cognition (Dall'Acqua et al., 2017; De Simoni et al., 2016; Palacios et al., 2017; Santhanam et al., 2019), which aligns with our results. Additionally, one study showed that lower connectivity within several networks, including the DMN and SN, was predictive for worse long‐term cognitive performance in patients with mild traumatic brain injury (Palacios et al., 2017). This differs from our findings since we found no relation between network strength and long‐term cognitive outcome. This difference might be explained by differences in underlying disease mechanisms and/or methodological differences. For example, we used the MoCA score and sum scores for memory, attention, and executive functioning as outcomes instead of individual test scores.

Functional connectivity in a small region in the precuneus was related to MoCA scores during hospital admission. This in line with previous literature on the relationship between connectivity in the precuneus and cognitive scores in patients with heart failure (Schroeter et al., 2023). This relationship in cardiac arrest patients can be explained by the important role of the precuneus in many complex cognitive tasks, and its high metabolic rate (Cavanna & Trimble, 2006; Dadario & Sughrue, 2023). Additionally, higher connectivity in regions in the right anterior temporoparietal junction was related to better cognition during hospital admission. These areas are known to play a role in attentional selection and social cognition (Krall et al., 2015; Mars et al., 2012; Martin et al., 2020; Wu et al., 2015).

The relation between functional connectivity within the networks and cognitive function probably weakens over time due to functional recovery in the weeks and months after the cardiac arrest, with neuroplasticity, reorganization, reweighting, and recruitment or use of alternative networks (Chen et al., 2010; Muñoz‐Cespedes et al., 2005). These processes are likely to influence both network strength and cognitive performance. We only measured network strength in the first weeks after cardiac arrest and not the evolution of network strength over time, while cognitive performance at 3 and 12 months was clearly better than during hospital admission.

Another possible factor influencing cognitive functioning after cardiac arrest is cognitive reserve or resilience (Hagberg et al., 2022). This includes, amongst others, age, education, lifestyle factors, chronic vascular changes, atrophy, and prior events. We did not take these factors into account in our current analyses since we first aimed to assess the possible value of resting‐state functional MRI scans. To eventually compose a multimodal prediction model for long‐term cognitive performance after cardiac arrest, these factors need consideration.

Since DMN connectivity is essential for recovery of consciousness (Threlkeld et al., 2018; Vanhaudenhuyse et al., 2010), we examined the integrity of the DMN in the patients who were still comatose during MRI scanning. Results showed that the network connectivity did not differ from patients who were conscious during scanning, and that the DMN could be identified in the comatose group. This implicates that the DMN was intact in the patient group who was still comatose during MRI scanning and regained consciousness later. This is in line with previous research into integrity of the DMN of comatose patients after cardiac arrest and traumatic brain injury who eventually regain consciousness (Keijzer, Lange, et al., 2022; Norton et al., 2012; Threlkeld et al., 2018). It is also consistent with work in primates, showing that the DMN could be detected in anesthetized animals (Vincent et al., 2007).

Strengths of this study include the prospective design, the use of a validated global cognitive screening tool and more detailed neuropsychological tests, measuring domain‐specific cognitive functioning, and the use of hypothesis‐free data‐driven analyses (ICA). There are also some limitations. First, we do not have cognitive outcome data available on all time points for all patients. Nevertheless, we decided to include all patients in our analyses to prevent bias towards patients with more favorable outcomes. The influence of missing data was minimized by analyzing the data using mixed‐effects models. Second, underlying structural abnormalities, e.g. white matter hyperintensities or diffusion restriction, could have confounded our analyses. We did not take these abnormalities into account, but a multimodal approach should be considered in future research. Third, the MRI interval ranged from 1.5 to 35 days: some patients were scanned between Days 2 and 10, while for others the MRI scan was postponed because of delirium. Since functional recovery is likely to be highly dynamic in the first weeks after cardiac arrest (Chen et al., 2010; Muñoz‐Cespedes et al., 2005), this might have influenced our results. Fourth, 15% of patients were sedated at time of the MRI. Administration of sedative drugs influences the connectivity in different resting‐state networks (Vincent et al., 2007; Greicius et al., 2008), and therefore also our relations. However, our sensitivity analysis showed that results were consistent after exclusion of the patients who were comatose during MRI scanning. Fifth, patients were included in three different hospitals using different MRI scanners. The influence was reduced as much as possible by harmonizing MRI protocols. To minimize the influence of MRI timing, sedative drug administration during the MRI, and study site, we included these variables as covariates in our mixed models.

## 5 CONCLUSION

Resting‐state functional connectivity in the DMN, SN, and DAN measured within the first weeks after cardiac arrest, during hospital admission, is related to short‐term global, but not long‐term global or domain‐specific cognitive performance of cardiac arrest survivors. These results do not support the value of functional connectivity within these RSNs for prediction of long‐term cognitive performance after cardiac arrest.

## CONFLICT OF INTEREST STATEMENT

The authors declare no conflicts of interest.

## Supporting information


**Data S1.** Supporting Information.

## Data Availability

The data that support the findings of this study are available from the corresponding author upon reasonable request.
