# Supplementary material for: Functional connectivity in resting‐state networks relates to short‐term global cognitive functioning in cardiac arrest survivors
Source: Hum Brain Mapp. 2024 Oct 24;45(15):e26769. doi: 10.1002/hbm.26769 (PMC11502408; doi:10.1002/hbm.26769)
Supplement: Supplementary file 1 — Data S1. Supporting Information. [file HBM-45-e26769-s001.docx]

# Functional connectivity in resting-state networks relates to short-term global cognitive functioning in cardiac arrest survivors

Marlous M.L.H. Verhulst^1,2^, Hanneke M. Keijzer^2^, Pauline C.W. van Gils^1,3,4^, Caroline M. van Heugten^4,5^, Frederick J.A. Meijer^6^, Bart A.R. Tonino^7^, Judith L. Bonnes^8^, Thijs S.R. Delnoij^9^, Jeannette Hofmeijer^1,2^, Rick C. Helmich^10,11^

1. Clinical Neurophysiology, TechMed Centre, University of Twente, Enschede, The Netherlands
2. Department of Neurology, Rijnstate Hospital, Arnhem, The Netherlands
3. Department of Psychiatry and Neuropsychology, School for Mental Health and Neuroscience, Maastricht University, Maastricht, The Netherlands
4. Limburg Brain Injury Center, Maastricht University, Maastricht, The Netherlands
5. Department of Neuropsychology and Psychopharmacology, Maastricht University, Maastricht, The Netherlands
6. Department of Medial Imaging, Radboud University Medical Center, Nijmegen, The Netherlands
7. Department of Radiology, Rijnstate Hospital, Arnhem, The Netherlands
8. Department of Cardiology, Radboud University Medical Center, Nijmegen, The Netherlands
9. Department of Cardiology, Maastricht University Medical Center+, Maastricht, The Netherlands
10. Radboud University Medical Centre, Donders Institute for Brain, Cognition and Behaviour, Neurology department, Centre of Expertise for Parkinson and Movement Disorders, Nijmegen, The Netherlands
11. Radboud University, Donders Institute for Brain, Cognition and Behaviour, Centre for Cognitive Neuroimaging, Nijmegen, The Netherlands

Key words

cardiac arrest, functional MRI, cognition, resting-state networks, functional connectivity

## Supplementary information

### fMRIPrep 21.0.1. pipeline

**Anatomical data preprocessing**

The T1-weighted (T1w) image was corrected for intensity non-uniformity (INU) with *N4BiasFieldCorrection* [61], distributed with ANTs 2.3.3 (RRID:SCR_004757, [62]), and used as T1w-reference throughout the workflow. The T1w-reference was then skull-stripped with a *Nipype* implementation of the *antsBrainExtraction.sh* workflow (from ANTs), using OASIS30ANTs as target template. Brain tissue segmentation of cerebrospinal fluid (CSF), white-matter (WM) and gray-matter (GM) was performed on the brain-extracted T1w using *fast* (FSL 6.0.5.1:57b01774, RRID:SCR_002823, [63]). Brain surfaces were reconstructed using *recon-all* (FreeSurfer 6.0.1, RRID: SCR_001847, [64]), and the brain mask estimated previously was refined with a custom variation of the method to reconcile ANTs-derived and FreeSurfer-derived segmentations of the cortical gray-matter of Mindboggle (RRID:SCR_002438, [65]). Volume-based spatial normalization to two standard spaces (MNI152NLin2009cAsym, MNI152NLin6Asym) was performed through nonlinear registration with *antsRegistration* (ANTs 2.3.3), using brain-extracted versions of both T1w reference and the T1w template. The following templates were selected for spatial normalization: *ICBM 152 Nonlinear Asymmetrical template version 2009c* (RRID:SCR_008796; TemplateFlow ID: MNI152NLin2009cAsym, [66]), *FSL’s MNI ICBM 152 non-linear 6^th^ Generation Asymmetric Average Brain Stereotaxic Registration Model* (RRID: SCR_002823; TemplateFlow ID: MNI152NLin6Asym, [67]).

**Functional data preprocessing**

For each of the BOLD runs found per subject (across all tasks and sessions), the following preprocessing was performed. First, a reference volume and its skull-stripped version were generated using a custom methodology of *fMRIPrep*. Head-motion parameters with respect to the BOLD reference (transformation matrices, and six corresponding rotation and translation parameters) are estimated before any spatiotemporal filtering using *mcflirt* (FSL 6.0.5.1:57b01774, [68]). The BOLD time-series (including slice-timing correction when applied) were resampled onto their original, native space by applying the transforms to correct for head-motion. These resampled BOLD time-series will be referred to as *preprocessed BOLD in original space*, or just *preprocessed BOLD*. The BOLD reference was then co-registered to the T1w reference using *bbregister* (FreeSurfer) which implements boundary-based registration [69]. Co-registration was configured with six degrees of freedom. Several confounding time-series were calculated based on the *preprocessed BOLD*: framewise displacement (FD), DVARS and three region-wise global signals. FD was computed using two formulations following Power (absolute sum of relative motions, [70]) and Jenkinson (relative root mean square displacement between affines, [68]). FD and DVARS are calculated for each functional run, both using their implementations in *Nipype* (following the definitions by Power et al. [70]). The three global signals are extracted within the CSF, the WM, and the whole-brain masks. Additionally, a set of physiological regressors were extracted to allow for component-based noise correction (*CompCor*, [71]). Principal components are estimated after high-pass filtering the *preprocessed BOLD* time-series (using a discrete cosine filter with 128s cut-off) for the two *CompCor* variants: temporal (tCompCor) and anatomical (aCompCor). tCompCor components are then calculated from the top 2% variable voxels within the brain mask. For aCompCor, three probabilistic masks (CSF, WM and combined CSF+WM) are generated in anatomical space. The implementation differs from that of Behzadi et al. [71] in that instead of eroding the masks by 2 pixels on BOLD space, the aCompCor masks are subtracted a mask of pixels that likely contain a volume fraction of GM. This mask is obtained by dilating a GM mask extracted from the FreeSurfer’s *aseg* segmentation, and it ensures components are not extracted from voxels containing a minimal fraction of GM. Finally, these masks are resampled into BOLD space and binarized by thresholding at 0.99 (as in the original implementation). Components are also calculated separately within the WM and CSF masks. For each CompCor decomposition, the k components with the largest singular values are retained, such that the retained components’ time series are sufficient to explain 50 percent of variance across the nuisance mask (CSF, WM, combined, or temporal). The remaining components are dropped from consideration. The head-motion estimates calculated in the correction step were also placed within the corresponding confounds file. The confound time series derived from head motion estimates and global signals were expanded with the inclusion of temporal derivatives and quadratic terms for each [72]. Frames that exceeded a threshold of 0.5 mm FD or 1.5 standardised DVARS were annotated as motion outliers. The BOLD time-series were resampled into standard space, generating a preprocessed BOLD run in MNI152NLin2009cAsym space. First, a reference volume and its skull-stripped version were generated using a custom methodology of fMRIPrep. Automatic removal of motion artifacts using independent component analysis (ICA-AROMA, [44]) was performed on the preprocessed BOLD on MNI space time-series after removal of non-steady state volumes and spatial smoothing with an isotropic, Gaussian kernel of 6mm FWHM (full-width half-maximum). Corresponding “non-aggressively” denoised runs were produced after such smoothing. Additionally, the ”aggressive” noise-regressors were collected and placed in the corresponding confounds file. All resamplings can be performed with a single interpolation step by composing all the pertinent transformations (i.e. head-motion transform matrices, susceptibility distortion correction when available, and co-registrations to anatomical and output spaces). Gridded (volumetric) resamplings were performed using *antsApplyTransforms* (ANTs), configured with Lanczos interpolation to minimize the smoothing effects of other kernels [73]. Non-gridded (surface) resamplings were performed using *mri_vol2surf* (FreeSurfer).

**Table S1**. Overview of neuropsychological tests within the three cognitive domains. Domain scores were calculated based on the mean z-score of the corresponding subtests.

| Domain | Test |
| --- | --- |
| Memory | RAVLT total recall trial 1-5  RAVLT delayed recall  RAVLT recognition |
| Attention | TMT-A (time)  Stroop-I (time)  Stroop-II (time)  Stroop-III (time) |
| Executive functioning | Short Raven  TMT B corrected for A  Stroop inference  Letter fluency (KOM) |

RAVLT = Rey Auditory Verbal Learning Test, TMT = Trail Making Test.

**Table S2.** Results of mixed effects regression model of connectivity strength within the default-mode network with sum z-scores for memory, attention, and executive functioning. This table shows the estimate with 95% confidence interval and p-value.

|  | **NPE** | | **Memory** | | **Attention** | | **Executive functioning** | |
| --- | --- | --- | --- | --- | --- | --- | --- | --- |
| *Predictors* | *Estimates* | *p* | *Estimates* | *p* | *Estimates* | *p* | *Estimates* | *p* |
| Intercept | -1.03 [-1.75; -0,31] | <0.01 | -1.44 [-2.29; -0.59] | <0.01 | -0.91 [-1.76; -0.06] | 0.04 | -0.78 [-1.63; 0.06] | 0.07 |
| DMN  Δ memory  Δ attention  Δ executive | 0.09 [-0.08; 0.25] | 0.30 | 0.13 [-0.06; 0.33]  -0.08 [-0.26; 0.11]  -0.05 [-0.24; 0.13] | 0.19  0.41  0.58 | 0.05 [-0.14; 0.25]  0.08 [-0.11; 0.26]  0.03 [-0.16; 0.21] | 0.59  0.41  0.79 | 0.08 [-0.12; 0.27]  0.05 [-0.13; 0.24]  -0.03 [-0.21; 0.16] | 0.43  0.58  0.79 |
| Study site  RAD  MUMC | 0.27 [-0.24; 0.79]  0.49 [-0.14; 1.13] | 0.30  0.13 | 0.28 [-0.24; 0.80]  0.50 [-0.14; 1.14] | 0.29  0.12 | 0.28 [-0.24; 0.80]  0.50 [-0.14; 1.14] | 0.29  0.12 | 0.28 [-0.24; 0.80]  0.50 [-0.14; 1.14] | 0.29  0.12 |
| Comatose | -0.17 [-0.78; 0.44] | 0.59 | -0.16 [-0.77; 0.45] | 0.61 | -0.16 [-0.77; 0.45] | 0.61 | -0.16 [-0.77; 0.45] | 0.61 |
| MRI time | -0.01 [-0.04; 0.03] | 0.62 | -0.01 [-0.04; 0.03] | 0.64 | -0.01 [-0.04; 0.03] | 0.64 | -0.01 [-0.04; 0.03] | 0.64 |

DMN = default-mode network.

**Table S3.** Results of mixed effects regression model of connectivity strength within the salience network with sum z-scores for memory, attention, and executive functioning. This table shows the estimate with 95% confidence interval and p-value.

|  | **NPE** | | **Memory** | | **Attention** | | **Executive functioning** | |
| --- | --- | --- | --- | --- | --- | --- | --- | --- |
| *Predictors* | *Estimates* | *p* | *Estimates* | *p* | *Estimates* | *p* | *Estimates* | *p* |
| Intercept | -1.22 [-1.94; -0.50] | <0.01 | -1.51 [-2.38; -0.65] | <0.01 | -1.16 [-2.03; -0.30] | <0.01 | -1.04 [-1.88; -0.19] | 0.02 |
| SN  Δ memory  Δ attention  Δ executive | 0.15 [-0.03; 0.33] | 0.11 | 0.16 [-0.05; 0.38]  -0.03 [-0.23; 0.16]  -0.01 [-0.20; 0.19] | 0.14  0.74  0.95 | 0.13 [-0.09; 0.34]  0.03 [-0.16; 0.23]  0.03 [-0.17; 0.22] | 0.24  0.74  0.79 | 0.16 [-0.06; 0.37]  0.01 [-0.19; 0.20]  -0.03 [-0.22; 0.17] | 0.15  0.95  0.79 |
| Study site  RAD  MUMC | 0.29 [-0.21; 0.80]  0.53 [-0.10; 1.16 | 0.26  0.10 | 0.30 [-0.21; 0.80]  0.54 [-0.09; 1.17] | 0.25  0.09 | 0.30 [-0.21; 0.80]  0.54 [-0.09; 1.17] | 0.25  0.09 | 0.30 [-0.21; 0.80]  0.54 [-0.09; 1.17] | 0.25  0.09 |
| Comatose | -0.08 [-0.69; 0.53] | 0.79 | -0.07 [-0.68; 0.54] | 0.81 | -0.07 [-0.68; 0.54] | 0.81 | -0.07 [-0.68; 0.54] | 0.81 |
| MRI time | -0.02 [-0.05; 0.02] | 0.38 | -0.01 [-0.05; 0.02] | 0.40 | -0.01 [-0.05; 0.02] | 0.40 | -0.01 [-0.05; 0.02] | 0.40 |

SN = salience network.

**Table S4.** Results of mixed effects regression model of connectivity strength within the default-mode network with MoCA scores during hospital admission, three months, and twelve months after cardiac arrest from the sensitivity analysis. This table shows the estimate with 95% confidence interval and p-value.

|  | **MoCA** | | **MoCA hospital** | | **MoCA 3 months** | | **MoCA 12 months** | |
| --- | --- | --- | --- | --- | --- | --- | --- | --- |
| *Predictors* | *Estimates* | *p* | *Estimates* | *p* | *Estimates* | *p* | *Estimates* | *p* |
| Intercept | 23.32 [20.59, 26.05] | <0.01 | 20.09 [16.89, 23.29] | <0.01 | 24.77 [21.40, 28.14] | <0.01 | 25.32 [22.05, 28.59] | <0.01 |
| DMN | 0.44 [-0.19, 1.07] | 0.17 | **0.82 [0.07, 1.57]** | **0.03** | 0.24 [-0.53, 1.02]] | 0.54 | 0.14 [-0.63, 0.91] | 0.72 |
| Study site  RAD  MUMC | 2.16 [-0.15, 4.48]  1.72 [-0.46, 3.91] | 0.07  0.12 | 1.61 [-0.70, 3.92]  1.66 [-0.56, 3.89] | 0.17  0.14 | 1.61 [-0.70, 3.92]  1.66 [-0.56, 3.89] | 0.17  0.14 | 1.61 [-0.70, 3.92]  1.66 [-0.56, 3.89] | 0.17  0.14 |
| MRI time | -0.10 [-0.20, 0.01] | 0.07 | -0.08 [-0.18, 0.03] | 0.16 | -0.08 [-0.18, 0.03] | 0.16 | -0.08 [-0.18, 0.03] | 0.16 |

DMN = default-mode network. Bold type font indicates statistical significance (p<0.05).

**Table S5.** Results of mixed effects regression model of connectivity strength within the salience network with MoCA scores during hospital admission, three months, and twelve months after cardiac arrest from the sensitivity analysis. This table shows the estimate with 95% confidence interval and p-value.

|  | **MoCA** | | **MoCA hospital** | | **MoCA 3 months** | | **MoCA 12 months** | |
| --- | --- | --- | --- | --- | --- | --- | --- | --- |
| *Predictors* | *Estimates* | *p* | *Estimates* | *p* | *Estimates* | *p* | *Estimates* | *p* |
| Intercept | 21.98 [19.21, 24.75] | <0.01 | 18.72 [15.33, 22.12] | <0.01 | 23.69 [20.16, 27.22] | <0.01 | 23.17 [19.93, 26.42] | <0.01 |
| SN | **0.83 [0.15, 1.52]** | **0.02** | **1.21 [0.37, 2.04]** | **<0.01** | 0.55 [-0.31, 1.41] | 0.21 | 0.72 [-0.08, 1.52] | 0.08 |
| Study site  RAD  MUMC | 2.00 [-0.25, 4.25]  1.80 [-0.30, 3.90] | 0.08  0.09 | 1.34 [-0.90, 3.58]  1.75 [-0.39, 3.90] | 0.24  0.11 | 1.34 [-0.90, 3.58]  1.75 [-0.39, 3.90] | 0.24  0.11 | 1.34 [-0.90, 3.58]  1.75 [-0.39, 3.90] | 0.24  0.11 |
| MRI time | **-0.11 [-0.21, -0.01]** | **0.03** | -0.09 [-0.19, 0.02] | 0.10 | -0.09 [-0.19, 0.02] | 0.10 | -0.09 [-0.19, 0.02] | 0.10 |

SN = salience network. Bold type font indicates statistical significance (p<0.05).

**Table S6.** Results of mixed effects regression model of connectivity strength within the default-mode network with sum z-scores for memory, attention, and executive functioning from the sensitivity analysis. This table shows the estimate with 95% confidence interval and p-value.

|  | **NPE** | | **Memory** | | **Attention** | | **Executive functioning** | |
| --- | --- | --- | --- | --- | --- | --- | --- | --- |
| *Predictors* | *Estimates* | *p* | *Estimates* | *p* | *Estimates* | *p* | *Estimates* | *p* |
| Intercept | -1.06 [-1.76; -0.36] | <0.01 | -1.47 [-2.31; -0.64] | <0.01 | -0.94 [-1.78; -0.10] | 0.03 | -0.81 [-1.64; 0.02] | 0.06 |
| DMN | 0.09 [-0.08; 0.25] | 0.30 | 0.13 [-0.06; 0.32] | 0.19 | 0.05 [-0.14; 0.25] | 0.59 | 0.08 [-0.12; 0.27] | 0.43 |
| Study site  RAD  MUMC | 0.27 [-0.24; 0.78]  0.52 [-0.11; 1.14] | 0.30  0.11 | 0.28 [-0.23; 0.79]  0.52 [-0.11; 1.15] | 0.29  0.10 | 0.28 [-0.23; 0.79]  0.52 [-0.11; 1.15] | 0.29  0.10 | 0.28 [-0.23; 0.79]  0.52 [-0.11; 1.15] | 0.29  0.10 |
| MRI time | -0.01 [-0.04; 0.03] | 0.65 | -0.01 [-0.04; 0.03] | 0.67 | -0.01 [-0.04; 0.03] | 0.67 | -0.01 [-0.04; 0.03] | 0.67 |

DMN = default-mode network.

**Table S7.** Results of mixed effects regression model of connectivity strength within the salience network with sum z-scores for memory, attention, and executive functioning from the sensitivity analysis. This table shows the estimate with 95% confidence interval and p-value.

|  | **NPE** | | **Memory** | | **Attention** | | **Executive functioning** | |
| --- | --- | --- | --- | --- | --- | --- | --- | --- |
| *Predictors* | *Estimates* | *p* | *Estimates* | *p* | *Estimates* | *p* | *Estimates* | *p* |
| Intercept | -1.25 [-1.93; -0.57] | <0.01 | -1.54 [-2.37; -0.71] | <0.01 | -1.19 [-2.02; -0.36] | <0.01 | -1.06 [-1.88; -0.25] | 0.01 |
| SN | 0.15 [-0.02; 0.33] | 0.09 | 0.17 [-0.04; 0.38] | 0.12 | 0.13 [-0.08; 0.34] | 0.22 | 0.16 [-0.05; 0.37] | 0.13 |
| Study site  RAD  MUMC | 0.29 [-0.21; 0.79]  0.54 [-0.08; 1.16] | 0.26  0.09 | 0.30 [-0.21; 0.80]  0.55 [-0.07; 1.16] | 0.25  0.08 | 0.30 [-0.21; 0.80]  0.55 [-0.07; 1.16] | 0.25  0.08 | 0.30 [-0.21; 0.80]  0.55 [-0.07; 1.16] | 0.25  0.08 |
| MRI time | -0.02 [-0.05; 0.02] | 0.39 | -0.01 [-0.05; 0.02] | 0.40 | -0.01 [-0.05; 0.02] | 0.40 | -0.01 [-0.05; 0.02] | 0.40 |

SN = salience network.

**Table S8.** Results of mixed effects regression models for the exploratory analysis of connectivity strength within the seven networks with MoCA scores during hospital admission, three months, and twelve months after cardiac arrest. This table shows the estimate with 95% confidence interval and p-value.

|  | **MoCA hospital** | | **MoCA 3 months** | | **MoCA 12 months** | |
| --- | --- | --- | --- | --- | --- | --- |
| *Predictors* | *Estimates* | *p* | *Estimates* | *p* | *Estimates* | *p* |
| VNL | -0.23 [-1.19; 0.74] | 0.64 | -0.27 [-1.32; 0.77] | 0.61 | -0.87 [-1.76; 0.03] | 0.06 |
| VNM | 0.01 [-0.70; 0.71] | 0.99 | 0.01 [-0.74; 0.76] | 0.98 | 0.03 [-0.65; 0.71] | 0.93 |
| SMN | 0.43 [-0.05; 0.91] | 0.08 | 0.15 [-0.36; 0.66] | 0.56 | 0.14 [-0.33; 0.61] | 0.57 |
| ECN | 0.35 [-0.38; 1.08] | 0.34 | 0.13 [-0.63; 0.89] | 0.74 | -0.09 [-0.76; 0.58] | 0.79 |
| CBN | -0.03 [-0.82; 0.75] | 0.94 | 0.15 [-0.18; 0.04] | 0.23 | -0.07 [-0.18; 0.04] | 0.23 |
| DAN | **0.96 [0.24; 1.69]** | **0.01** | 0.27 [-0.48; 1.02] | 0.48 | 0.17 [-0.53; 0.87] | 0.63 |
| FPN | 0.35 [-0.83; 1.53] | 0.56 | -0.19 [-1.41; 1.03] | 0.76 | -0.11 [-1.22; 1.00] | 0.84 |

CBN = cerebellar network, DAN = dorsal attention network, ECN = executive control network, FPN = frontoparietal network, SMN = sensorimotor network, VNL = lateral visual network, VNM = medial visual network

**Table S9.** Results of mixed effects regression models for the exploratory analysis of connectivity strength within the seven networks with MoCA scores during hospital admission, three months, and twelve months after cardiac arrest. This table shows the estimate with 95% confidence interval and p-value.

|  | **Memory** | | **Attention** | | **Executive functioning** | |
| --- | --- | --- | --- | --- | --- | --- |
| *Predictors* | *Estimates* | *p* | *Estimates* | *p* | *Estimates* | *p* |
| VNL | -0.19 [-0.43; 0.05] | 0.12 | 0.04 [-0.20; 0.28] | 0.75 | -0.08 [-0.32; 0.16] | 0.50 |
| VNM | -0.04 [-0.24; 0.16] | 0.68 | 0.12 [-0.08; 0.31] | 0.24 | 0.05 [-0.14; 0.24] | 0.58 |
| SMN | 0.01 [-0.11; 0.14] | 0.83 | 0.06 [-0.06; 0.19] | 0.31 | 0.06 [-0.06; 0.18] | 0.34 |
| ECN | 0.03 [-0.14; 0.21] | 0.73 | 0.12 [-0.05; 0.30] | 0.17 | 0.03 [-0.14; 0.21] | 0.71 |
| CBN | -0.14 [-0.35; 0.07] | 0.19 | -0.09 [-0.30; 0.12] | 0.41 | -0.06 [-0.25; 0.14] | 0.57 |
| DAN | 0.09 [-0.09; 0.28] | 0.33 | 0.17 [-0.01; 0.36] | 0.07 | 0.10 [-0.08; 0.29] | 0.28 |
| FPN | 0.11 [-0.18; 0.40] | 0.46 | 0.17 [-0.12; 0.46] | 0.25 | 0.10 [-0.19; 0.39] | 0.48 |

CBN = cerebellar network, DAN = dorsal attention network, ECN = executive control network, FPN = frontoparietal network, SMN = sensorimotor network, VNL = lateral visual network, VNM = medial visual network


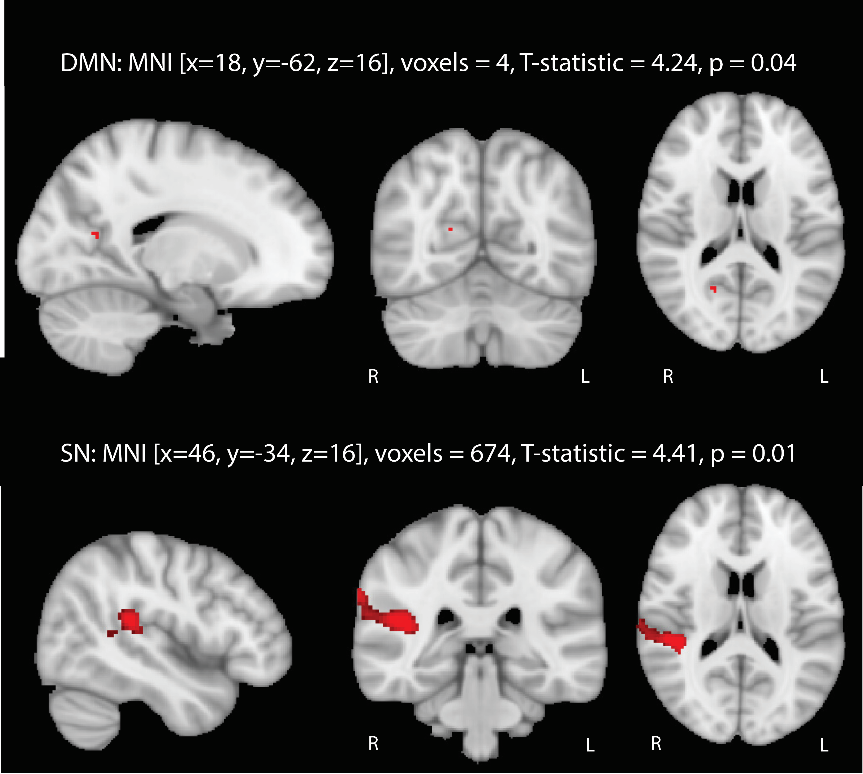


**Figure S1**. Results of voxel wise analyses for the correlation between voxel wise connectivity and MoCA scores. Results are TFCE FWE-corrected (p < 0.05). Highest T-statistic and most significant p-value shown. DMN = default-mode network, SN = salience network.


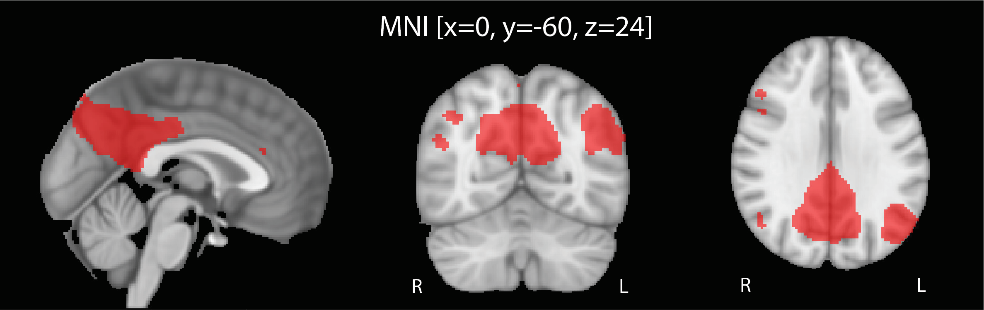


**Figure S2**. Default-mode network as identified in the group of patients who were comatose during MRI scanning (n=12).
